# Simultaneous Development of a Multidimensional Fatalism Measure in English and Spanish

**DOI:** 10.1007/s12144-014-9272-z

**Published:** 2014-09-21

**Authors:** Oscar A. Esparza, John S. Wiebe, Juan Quiñones

**Affiliations:** Universidad Autonoma de Ciudad Juarez, University Av. And H. Colegio Militar, Ciudad Juarez, Chihuahua, C.P. 32300 Mexico; The University of Texas at El Paso, El Paso, TX USA

**Keywords:** Fatalism, Measurement invariance, Factor analysis

## Abstract

Fatalism has been shown to predict several health behaviors, but researchers often find inconsistent results for the same behaviors across studies. This may be partially attributable to the diversity of fatalism measures that have been used in previous studies. A review of the literature revealed 51 different scales, all purported to measure fatalism, but often with heterogeneous content (Esparza 2005). A study done by Esparza (2005) retrieved 29 scales, including the most frequently used scales, and performed an exploratory factor analysis, obtaining as a result five factors: fatalism, helplessness, internality, luck, and divine control. The purpose of this study was to develop a multidimensional fatalism scale based on the previous findings by Esparza (2005). This scale was developed simultaneously in English and Spanish in order to linguistically “decenter” item content. The factor structure was cross-validated and measurement invariance was assessed across language versions. According to the measurement invariance analysis, this test is invariant across English and Spanish in its factor structure, loadings, variances, and covariances. This study results suggest that this scale may be used interchangeably in both English and Spanish.

Fatalism has been a construct of increasing interest in personality and health psychology over the past thirty years. A literature review performed with the PsycINFO database using the term “fatalism” returned 80 published studies from 1985 to 1994, 169 studies from 1995 to 2004, and 334 studies from 2005 to 2014. However, in this extensive body of work, fatalism has been defined and operationalized in many different ways. For example, it has been defined as the belief that outcomes are predetermined by external forces (Ross et al. 1983). Futa et al. (2001) defined it as the acceptance of one’s situation. Fatalism has also been interpreted as a potentially adaptive response to uncontrollable life situations experienced by minorities (Parker and Kleiner 1966). Wheaton (1983) stated that fatalism is a tendency to believe in the efficacy of environmental rather than personal forces in understanding the causes of life outcomes, including both “success” and “failure” outcomes. Scheier and Bridges (1995) focused on the pessimistic aspect of fatalism, claiming that fatalism and pessimism share a common core that involves negative expectations regarding future outcomes. According to these definitions, fatalism has been variously characterized as external locus of control, belief in predetermination, acceptance of reality, or a coping skill. While the term has been usually imbued with negative connotations, there have been at least some definitions that suggest a positive perspective.

Merriam-Webster’s collegiate dictionary (1993) defined fatalism as “the doctrine that all events are subject to fate or inevitable predetermination; the acceptance of all things and events as inevitable; submission to fate” (p. 517). However, there has not been a comprehensive theory of fatalism in the psychology literature. In an initial effort to identify some structure in the field, Acevedo (2005a) reviewed several findings regarding fatalism. He noted that from the sociological perspective, fatalism could be viewed in the context of theories by Durkheim and Marx (Acevedo 2005b) who focused on the over-regulation by social structures, though Acevedo (2005a) also proposed a fatalism theory that included a cognitive factor. He held that fatalism could be the result of social structures, like slavery, but it could also be a cognitive orientation in people.

Acevedo (2005a) distinguished between two types of fatalism: structural and cosmological. Structural fatalism was defined as “a sense of powerlessness that results from a combination of over regulation combined with a lack of exit option into the collective body in which the subject lacks the necessary voice and/or exit option to alter their social position, status, rank, or living conditions” (Acevedo 2005a, p. 75). Structural fatalism could result from feeling powerless due to social structures, like slavery or poverty, which overregulate the lives of people, but structural fatalism could also involve powerlessness due to a perceived lack of self-efficacy, which has been associated with lack of control or mastery (Acevedo 2005a). Cosmological fatalism was defined as “the extent to which a given individual or collective group grants control and decision making authority over life’s outcomes to cosmological or metaphysical powers” (Acevedo 2005a, p. 73). Most frequently, this type of fatalism has involved a voluntary resignation of aspects of one’s life to divine control. Cosmological fatalism has involved “internalization” of one’s fate, while structural fatalism has only involved “acceptance,” rather than internalization of fate. Acevedo emphasized that the types of fatalism he described are different, but not mutually exclusive.

Acevedo’s two types of fatalism parallel, to some extent, work on secondary control, initially outlined by Rothbaum et al. (1982), and more recently reviewed and refined by Morling and Evered (2006). Morling and Evered defined secondary control as “…adjust[ing] some aspect of the self and accept[ing] circumstances as they are” (2006, p. 272). They distinguished between “adjustment” of the self to improve fit with environmental circumstances (a term closely related to Acevedo’s concept of “internalization”), and mere “acceptance” of events, suggesting that both characteristics were necessary to achieve secondary control. Thus, it would seem that Acevedo’s conceptualization of fatalism included secondary control expectations, but focused primarily on acceptance of current and future circumstances, without requiring that adjustment or internalization took place. While Acevedo’s work has begun to outline important theoretical distinctions in the area of fatalism, the area has remained largely undirected by theory, with many researchers using various, and often little-overlapping, measures to assess what they term “fatalism.”

## Different Fatalism Scales

A literature search done by Esparza (2005), using the PsychInfo database and the search terms “fatalistic” and “fatalism,” revealed 51 different scales, all explicitly purported by researchers to measure fatalism. Some scales were used quite often, like Rotter’s Internal-External Locus of Control Scale (Rotter 1966), but other scales were constructed and used only for a single study. In addition, some researchers have simply used measures of other constructs as proxies for fatalism. Many researchers have measured fatalism as locus of control (e.g., Joiner et al. 2001; Schedlowski et al. 1995; Wade 1996). Fatalism has been conceptualized as a coping skill in different scales (e.g., Akechi et al. 2001; Classen et al. 1996; Moneyham et al. 1997). Measures of mastery have included fatalism in their subscales (e.g., Green and Rodgers 2002; Neff 1993; Pearlin and Schooler 1978). Other scales and articles have equated fatalism with learned helplessness (Clarke et al. 1982), lack of activism (Chandler 1979; Farris and Glenn 1976), world views (Goodwin et al. 1999; Peck 1981), facets of personality (Wheaton 1983), belief in a just world (Lipkus 1991), perceptions and attitudes about safety (Rudomo and Hale 2003; Williamson et al. 1997), cultural values or constructs (Cuéllar et al. 1995a; b; Sanchez and Garriga 1996), pessimism (Scheier and Bridges 1995), death threat (Moore and Neimeyer 1991), and views and expectations for the future (De Brabander et al. 1999; Somlai et al. 2000).

Recently, the most used fatalism scale has been the Powe fatalism Inventory (Powe 1995). Several cancer studies have used this scale (e.g., Davis et al. 2002; Dettenborn et al. 2004; Farmer et al. 2007; Gonzalez 2007; Gorin 2005; Greiner et al. 2005; Gullatte et al. 2010; Lawsin et al. 2006; Magai et al. 2004; Mayo et al. 2001; Pines 2002; Powe et al. 2006; Russell et al. 2006; Talbert 2007), that has assessed several components of cancer fatalism including belief in divine control and predetermination, pessimism, resignation, and the perceived inevitability of death associated with a cancer diagnosis. All of the items of this scale were related to cancer, for example “I believe if someone gets cancer, it was meant to be”. However, although these constructs have been described by researchers as representing fatalism, it is unclear whether they have had any semantic similarity. In their review of the measurement of fatalism, Abraído-Lanza et al. (2007) mentioned that there has been a lack of established and reliable scales, limited evidence of the validity of existing measures and that the different scales may have tapped different fatalism constructs, suggesting a need to develop complex, valid and reliable fatalism measures.

There have been two new fatalism scales developed recently, but they are specific to health beliefs and diabetes. Shen et al. (2009), developed a scale in which they conceptualized fatalism as a set of health beliefs. Their scale was composed of 20 items divided in three dimensions: predetermination, luck and pessimism. These factors are similar to the factors proposed in the present study but the difference is that Shen et al. (2009) adapted all of their items to health beliefs, for example, “I will get diseases if I am unlucky”. Egede and Ellis (2010) developed a 12-item diabetes fatalism scale with three factors: emotional distress, religious and spiritual coping and perceived self-efficacy. All of their items were related to diabetes, for example, “I get frustrated with having to live with diabetes”.

## Analysis of Several Fatalism Scales

In an attempt to bring order to the construct of fatalism and its measures, Esparza (2005) did a literature search and found 51 different scales, all purported to measure fatalism. Twenty-nine of the most commonly used scales were used to analyze the construct. An exploratory factor analysis was performed on 239 items that were obtained from the 29 fatalism scales. Items loaded onto five main factors. The content of the first factor was labeled “fatalism” because it most clearly reflected the belief that events are fixed in advance. Examples of items that loaded on this factor were “When something bad happens, I accept it as unavoidable,” and “If bad things happen, it is because they were meant to be.” This factor appeared to define the core of the fatalism construct and related more closely than the others to the definition given by Merriam-Webster’s collegiate dictionary (1993)—“a doctrine that events are fixed in advance so that human beings are powerless to change them” (p. 517).

Items from the second factor were related to perceptions of helplessness and pessimism, and thus the factor was labeled helplessness. Examples of items that loaded on this factor were “Every time I try to get ahead, something or somebody stops me,” and “I often feel helpless in dealing with the problems of life.” Items that loaded onto the third factor were all related to expectancies of internal control over important life events. They were all reverse coded in an effort to measure fatalism. Examples of items that loaded on this factor were “When I get what I want, it's usually because I worked hard for it,” and “What happens to me in the future mostly depends on me.” Almost all items that loaded on the fourth factor included the words “luck” or “fortune.” Examples of items that loaded on the fourth factor were “Luck plays a big part in determining how soon I will recover from an illness,” and “Many of the unhappy things in people's lives are partly due to bad luck.” All of the items that loaded on the fifth factor included the word “God.” Examples of items that loaded on this factor were “Whether or not my health improves is up to God,” and “Everything that happens is part of God's plan.” This factor, dubbed “divine control,” was highly internally consistent. Finally, there were 108 items that did not load on any of the five factors, for example “Whenever I don’t feel well, I should consult a medically trained professional” and “I’ve had a good life, what’s left is a bonus.”

Items from these scales did not measure a single fatalism construct but rather five different, but correlated, factors. The lowest correlations were between the factors of helplessness and divine control (*r* = 0.05), the factors of externality and divine control (*r* = 0.10), the factors of luck and divine control (*r* = 0.20), and the factors of ineluctable destiny and externality (*r* = 0.27). The highest correlation was between the factors of ineluctable destiny and helplessness (*r* = 0.48). The divine control factor had small correlations with most of the other scales. In addition, many items did not load on the five factors. The findings suggested a need to elaborate a fatalism instrument that brings unity in its measure. This type of scale would help us advance the understanding of the construct of fatalism and its relationships to other variables.

## The Present Study

The present study was undertaken to develop a new multidimensional measure of fatalism and related constructs informed by Esparza (2005). In the development of new instruments there is a risk of ethnocentric bias that may threaten cross-cultural validity (Tanzer 2005). The term “centering” refers to the origination of a scale in one language and culture, embedding it in that linguistic and cultural context to an extent that it is difficult or inappropriate to transfer the scale to a new context. Tanzer (2005) pleads for simultaneous development of scales to maximize cultural and linguistic “decentering,” in an attempt to reduce biases across cultures from the beginning. The process of simultaneous development involves constructing items for a scale in two languages at the same time. Because of the likelihood that a new fatalism measure would be used in cross-cultural and multilingual research, a simultaneous development approach was used in the current study, and measurement invariance analyses were employed to assess the equivalence of the two versions of the instrument.

## Method

### Participants

The sample consisted of 791 participants recruited from Introduction to Psychology classes at the University of Texas at El Paso. Participants were 64.6 % females and 35.4 % males, with a mean age of 20.34 (σ = 4.65). Most of the sample was Hispanic (86.2 %), with non-Hispanic White participants making up 7.5 % of the sample. In regard to marital status, 90.5 % were single and 7 % were married or living with a romantic partner. The mean socioeconomic status for the head of household, measured by the two-factor Hollingshead socioeconomic status index, was 42.58, which reflects a social stratum comprising medium business, minor professional, and technical workers (Hollingshead 1965). The sample was divided randomly into two subsamples: One of the subsamples was used for the exploratory factor analysis (*n* = 400) and the second subsample for the confirmatory factor analysis (*n* = 391). There were 513 participants who answered the fatalism items in English during the first administration and 278 in Spanish.

In discussing power analyses for factor analysis, MacCallum et al. (1999) note that sample size should be related to item communalities, number of factors, and number of items per factor. MacCallum et al. (1999) suggest that “if results show a relatively small number of factors and moderate to high communalities, then the investigator can be confident that obtained factors represent a close match to population factors, even with moderate to small sample sizes” (p. 97). In this study, communalities for the exploratory factor analysis ranged from 0.63 to 0.90 and the items were written to load on the expected factors.

### Materials

#### Demographics

Participants were asked about their age, gender, ethnicity, and proficiency in English and Spanish.

#### Two-Factor Hollingshead Socioeconomic Status Index

This index is typically computed from occupation and education of the participant (Hollingshead 1965). However, because participants in this study were students, they were asked to provide the information on the current head of their household. Hollingshead (1975) reported that the two factors accounted for 98 % of the variance in sociologist’s ratings of social status, suggesting that the index has adequate construct and content validity. Hollingshead ratings were completed by a rater (the first author) who was trained to an inter-rater reliability correlation of *r* = 0.98. Scores ranged from 11 to 77, with smaller scores corresponding to higher socioeconomic status.

#### Acculturation Rating Scale for Mexican Americans II (ARSMA-II)

This instrument measures the construct of acculturation to the Mexican and the Anglo cultures. The ARSMA-II contains two independent scales that measure Mexican Orientation and Anglo Orientation separately. The Mexican Orientation Subscale (MOS) is composed of 17 items and the Anglo Orientation Subscale (AOS) is composed of 13 items that are answered on a 5-point Likert scale ranging from “Almost Always/Extremely Often” to “Not at all.” Higher scores on the subscales mean greater identification with the culture each is measuring. The MOS has an internal consistency score of α = 0.88 and the AOS has an internal consistency of α = 0.83 (Cuéllar et al. 1995a; b).

#### Attributional Style Questionnaire (ASQ)

The Attributional Style Questionnaire was developed to assess dispositional attributions hypothesized to lead to learned helplessness and depressed mood (Peterson et al. 1982). The ASQ measures three dimensions: Internality, Stability, and Globality. For purposes of this study, only the Internality dimension was used to analyze its relationship with the internality factor of our fatalism scale. Higher scores are related to more internal locus. Although widely used by researchers in the past, the internality scale has the lowest internal reliability of the three dimensions, with a Cronbach alpha of 0.50 for positive events and 0.46 for negative events (Peterson et al. 1982).

#### Belief in Good Luck Scale (BIGL)

This measure reflects a belief in a stable and personal good fortune, distinct from the concepts of optimism, self-esteem, or locus of control (Darke and Freedman 1997). The instrument is composed of 12 items with a five-point Likert type response scale ranging from “Strongly Disagree” (1) to “Strongly Agree” (5). Higher scores mean participants believe more strongly that life has been good to them—that they have good luck. The internal consistency of this scale is α = 0.85, and the 1–2 month test-retest reliability is *r* = 0.63 (Darke and Freedman 1997).

#### Patient Health Questionnaire (PHQ-9)

This is a self-report measure from the Prime-MD (Spitzer et al. 1994) that consists of nine items assessing self-report depressive symptoms from the DSM-IV from 0 (not at all) to 3 (nearly every day). According to the authors, PHQ-9 scores of 5, 10, 15, and 20 represented mild, moderate, moderately severe, and severe depression, respectively. The reported internal reliability (alpha) of the PHQ-9 has ranged from 0.86 to 0.89 (Kroenke et al. 2001).

#### Life Orientation Test- Revised (LOT-R)

This instrument measures optimism for the future. This is a six-item measure with four filler items. Higher values imply optimism. The answer format is a five-point Likert type scale ranging from “I agree a lot” (1), to “I disagree a lot” (5). Its Cronbach alpha is 0.82 (Scheier and Carver 1992) and its 28-month test-retest reliability is *r* = 0.79 (Scheier et al. 1994).

#### Duke Religion Index (DRI)

This is a five-item instrument of religiosity with internal consistency of α = 0.91 (Storch et al. 2004). The scale was developed to assess beliefs, practices, and personal devotions.

### Design and Procedure

This study was approved by the Institutional Review Board for Human Subjects at UTEP. Participants rendered informed consent, and group seating and data collection efforts were structured to maximize confidentiality and anonymity of responses. For the first phase, items were generated in English and Spanish using the simultaneous development approach proposed by Tanzer (2005). This procedure involves a “committee approach” which includes bilingual people with the experience of both cultures and with experience in mainstream psychology, psychometrics, test construction techniques, cultural psychology, cross-cultural psychology, and linguistics (Tanzer 2005). When items are written in two languages simultaneously, idioms specific to one language or culture are generally avoided, leading to decentering of the measure and easier translation to other linguistic and cultural contexts.

For the second phase of the study, an empirical item selection approach was applied. Exploratory factor analysis was used to test the hypothesized factor structure and obtain the items with the highest loadings on each of the factors. Once items were selected, the factor structure was cross-validated through a confirmatory factor analysis with a second sample. The confirmatory factor analysis assessed model fit of the new multidimensional fatalism measure.

The third step was to analyze measurement invariance and structural invariance across the English and Spanish versions of the fatalism measure (Byrne et al. 1989). Measurement invariance included invariance of factor loadings, regression intercepts, and error/uniqueness variances, while structural invariances included invariance of factor variance-covariance structures and factor means (Byrne et al. 1989).

Convergent and discriminant validities were assessed in the fourth phase. From literature review, a list of brief and well-established measures conceptually associated with the hypothesized factors was obtained. Pessimism (the LOT-R) and depression (the PHQ-9) were used to assess the convergent validity of the helplessness factor (Anderson 1990; Burns and Seligman 1991; Luten et al. 1997: Peterson and Vaidya 2001). The internal attribution subscale of the ASQ was used to assess convergent validity of the internality factor The BIGL was selected as the most conceptually similar to the luck factor. The divine control factor was paired with the Duke Religion Index. The fatalism factor has, in past research, been the first and strongest factor from items used in other fatalism scales; it is the factor that best reflects the core concept of fatalism as defined by the Merriam-Webster’s collegiate dictionary (1993). Since no existing measure assesses that single factor, it was decided to only assess discriminant validity of this factor.

## Results

### Item Generation and Selection

Items were elaborated by two bilingual/bicultural content experts in both English and Spanish simultaneously for each factor of the five factors identified in the first phase. Items were required to have a similar meaning in English and Spanish using a comparable vocabulary and avoiding idioms. Each expert created a list of 20 items per factor based on the factor definitions and sample items from the study done by Esparza (2005). After eliminating identical items from this original list of 40 items per factor, the fatalism factor had 31 items, the helplessness factor had 36 items, the internality factor had 30 items, the luck factor had 37 items, and the divine control factor had 37 items. To further reduce the number of items (and the required sample size for factor analysis) the authors chose the best 18 items per factor, based on face validity. Items that addressed the construct in a broad manner were kept (e.g., If bad things happen to me in life, it is because of God's will), and items with more topic- specific content were rejected (e.g., It doesn't matter who is in political power, since it's God's will what happens in a country). The pool of items was then revised in both languages by a bilingual group of graduate students and undergraduate students in psychology. A final revision of the translations was done by a certified translator.

The readability of the scales in English and Spanish was analyzed. For the English form, the Flesh-Kincaid Grade Level (FKGL) value was 5.67, indicating a reading level of 5th grade. The Flesh Reading Ease (FRE) value was 79.18, indicating a reading level of a 7th grade. For the Spanish form, the Huerta Reading Ease (HRE) index was 91.73, indicating a reading level of 5th grade.

Two different versions (Form A and Form B) of the fatalism questionnaire were generated, with items ordered randomly to control for order effects. Order effects were assessed following the procedure by Panter et al. (1992). Invariance was assessed across Form A and Form B, and no order effects were found. The sample size for Form A was 398 and for Form B it was 393.

### Exploratory Factor Analysis and Cross-Validation—Confirmatory Factor Analysis

#### Missing Data

There were missing data on the fatalism measures. Out of the 71,190 data values from the fatalism measures, 134 (0.19 %) were missing. Since all participants answered most or all of the items, and since the percentage of missing data was low, it was decided to impute missing data. Data imputation was performed using the expectation maximization algorithm (Dempster et al. 1977) with the computer program NORM 2.03 (Schafer 2000).

#### Exploratory Factor Analysis

Our initial group of 862 participants was randomly divided into two samples. The exploratory factor analysis was performed with all 90 items and with a sample size of 400. Five factors were extracted using generalized least squares with a Promax rotation. Eigenvalues for the five factors ranged from 16.21 for the divine control factor to 2.22 for the fatalism factor. The percent of the variance explained by each factor ranged from 18.01 % for the divine control factor to 2.47 % for the fatalism factor. Items loaded on the expected factors. Based on the factor loadings, six items per factor were selected for scale inclusion. The selected items were those with the highest loadings that did not have a higher loading on other factors (Table 1). Loadings for the selected items ranged from 0.50 to 0.88 and the communalities of the selected items ranged from 0.63 to 0.90.

**Table 1:** Factor loadings and communalities for exploratory factor analysis of fatalism scale

Time	Factor loadings					Communality
	1	2	3	4	5	h^2^
Divine control						
Everything that happens is part of God’s plan.	0.88	−0.05	0.04	−0.15	0.33	0.90
Everything that happens to a person was planned by God.	0.85	0.02	0.08	−0.13	0.32	0.86
Whatever happens to me in my life, it is because that is the way God wanted it to happen.	0.83	0.03	0.11	−0.15	0.39	0.83
God controls everything good and bad that happens to a person.	0.82	0.05	0.17	−0.21	0.28	0.85
God has a plan for each person, and you cannot change his plan.	0.81	−0.04	0.05	−0.13	0.40	0.84
No matter how much effort I invest into doing things, at the end, God’s decisions will prevail.	0.80	0.00	0.13	−0.20	0.31	0.80
Luck						
When I get what I want, it’s usually because I’m lucky.	0.05	0.76	0.36	−0.25	0.03	0.71
How successful people are in their job is related to how lucky they are.	0.12	0.72	0.41	−0.23	0.08	0.77
Some people are simply born being lucky.	0.06	0.71	0.27	−0.17	0.07	0.70
When good things happen to people, it is because of good luck.	0.10	0.71	0.32	−0.16	0.22	0.72
The really good things that happen to me are mostly because of luck.	0.12	0.69	0.34	−0.15	0.23	0.77
Luck does not exist	0.01	−0.67	−0.06	0.09	−0.18	0.77
Helplessness						
I feel that nothing I can do will change things.	0.27	0.27	0.69	−0.48	0.20	0.73
Sometimes I feel there is nothing to look forward to in the future.	0.13	0.24	0.68	−0.33	0.15	0.73
I feel that I do not have any control over the things that happen to me.	0.30	0.27	0.64	−0.46	0.34	0.73
No matter how hard I try, I still cannot succeed in life.	0.14	0.23	0.62	−0.33	0.03	0.65
I often feel overwhelmed with problems, since I do not have any control over solving these problems.	0.15	0.32	0.61	−0.27	0.19	0.66
There’s nothing I can do to succeed in life, since one’s level of success is determined when one is born.	0.33	0.26	0.57	−0.31	0.09	0.68
Internality						
I feel that when good things happen, they happen as a result of my own efforts.	−0.22	−0.27	−0.37	0.70	−0.03	0.74
What happens to me in the future mostly depends on me.	−0.27	−0.15	−0.33	0.68	−0.09	0.73
My life is determined by my own actions.	−0.18	−0.20	−0.31	0.65	−0.03	0.65
What people get out of life is always due to the amount of effort they put into it.	−0.07	−0.29	−0.27	0.62	0.14	0.66
What happens to me is a consequence of what I do.	−0.22	−0.29	−0.28	0.62	0.00	0.67
I can do almost anything if I really want to do it.	−0.11	−0.22	−0.39	0.61	0.09	0.68
Fatalism						
I have learned that what is going to happen will happen.	0.46	0.22	0.29	−0.11	0.69	0.75
If something bad is going to happen to me, it will happen no matter what I do.	0.40	0.15	0.31	−0.15	0.61	0.67
If bad things happen, it is because they were meant to happen.	0.47	0.14	0.23	−0.14	0.58	0.66
There is no sense in planning a lot; if something good is going to happen, it will.	0.26	0.22	0.42	−0.20	0.54	0.66
Life is very unpredictable, and there is nothing one can do to change the future.	0.26	0.09	0.32	−0.13	0.51	0.63
People die when it is their time to die and there is not much that can be done about it.	0.32	−0.10	0.02	0.02	0.50	0.64
Other items						
Only God knows, and only He will determine what will become of our lives.	0.80	−0.01	0.14	−0.15	0.40	0.83
It doesn’t do any good to try to change the future because the future is in the hands of God.	0.75	0.05	0.33	−0.22	0.33	0.81
God is in control of my health.	0.74	0.02	0.11	−0.21	0.18	0.77
There is nothing I can do to change the will of God.	0.73	−0.07	0.04	−0.09	0.39	0.74
God is directly responsible for my health getting better or worse.	0.72	0.16	0.26	−0.24	0.20	0.76
It doesn’t matter what I want to do with my life, since God has total control over what happens.	0.70	0.07	0.36	−0.25	0.16	0.78
If bad things happen to me in life, it is because of God’s will.	0.70	0.16	0.27	−0.24	0.27	0.73
God will protect me from any harm.	0.67	0.04	0.04	−0.01	0.19	0.72
It really doesn’t help to plan things because everything is in God’s hands.	0.63	0.12	0.38	−0.18	0.32	0.67
God will take care of me even if I do not take care of myself.	0.54	0.18	0.20	−0.11	0.20	0.66
There’s no sense in getting checked by the doctor since my health depends only on God’s will.	0.41	0.24	0.35	−0.22	0.12	0.63
Trusting to faith has never worked out as well for me as taking action	−0.36	0.02	0.02	0.22	−0.07	0.52
People who are lucky always get what they want out of life.	0.07	0.69	0.34	−0.11	0.00	0.73
If someone is in a position of power, it’s because he/she was born lucky.	0.12	0.68	0.47	−0.27	−0.04	0.74
Many of the unhappy things in people’s lives are partly caused by bad luck.	0.01	0.60	0.35	−0.26	0.05	0.64
Accidents usually happen to people who have bad luck.	0.10	0.58	0.39	−0.21	0.04	0.63
If a person gets sick, it’s usually related to his or her bad luck.	0.11	0.57	0.47	−0.31	−0.15	0.67
I don’t believe that luck has anything to do with what happens to a person.	0.04	−0.57	−0.09	0.13	0.09	0.71
Good luck is more important than hard work to succeed in life.	0.12	0.50	0.45	−0.29	−0.01	0.65
Becoming successful has to do with hard work; luck has little or nothing to do with it.	−0.01	−0.47	−0.23	0.34	−0.01	0.62
It is impossible for me to believe that luck plays an important role in my life.	−0.01	−0.47	−0.01	0.12	−0.12	0.69
It is impossible for me to believe that luck plays an important role in my life.	−0.07	−0.39	0.02	0.10	−0.18	0.62
In my case, getting what I want has little or nothing to do with luck.	−0.03	−0.39	−0.12	0.26	0.04	0.43
In most cases, I do not depend on luck when I decide to do something.	−0.05	−0.26	−0.12	0.20	0.00	0.38
I feel that when I make a mistake, there’s very little I can do to correct it.	0.18	0.24	0.55	−0.25	0.24	0.65
I often feel powerless when dealing with the problems of life.	0.15	0.18	0.55	−0.15	0.32	0.62
Every time I try to make progress, something or somebody stops me.	0.12	0.31	0.54	−0.22	0.16	0.67
When something bad happens, I feel nothing can be done about it.	0.18	0.21	0.52	−0.25	0.43	0.61
Sometimes I feel that I don’t have enough control over the direction my life is taking.	0.14	0.18	0.50	−0.13	0.27	0.68
There’s really nothing one can do to change one’s health, since a person has no control over what happens to his or her health.	0.28	0.26	0.46	−0.18	0.23	0.61
If someone was not destined to succeed, no matter how much effort they give, they will not succeed.	0.30	0.32	0.45	−0.37	0.15	0.58
There is really no way I can solve some of the problems I have.	0.05	0.14	0.45	−0.24	0.19	0.58
I see bad things happening in my future.	−0.02	0.13	0.43	−0.16	0.04	0.61
Unfortunately, no matter how hard we try, our worth frequently passes unrecognized	0.14	0.14	0.41	−0.18	0.24	0.51
There’s nothing I can do to change the things I do not like in my work.	0.02	0.20	0.39	−0.17	0.07	0.56
If I take the right actions, I can stay healthy.	−0.09	−0.13	−0.22	0.61	−0.13	0.69
I think that people can get what they want if they keep trying.	−0.08	−0.23	−0.34	0.57	−0.01	0.55
When I get what I want, it’s usually because I worked hard for it.	−0.07	−0.14	−0.27	0.56	0.04	0.64
I am in control of my health.	−0.22	−0.11	−0.19	0.51	−0.12	0.68
If I take care of myself, I can avoid illnesses.	−0.12	−0.07	−0.17	0.51	−0.13	0.63
I think that planning is very important in determining a person’s future.	−0.06	−0.21	−0.31	0.48	−0.10	0.60
In the long run people receive the respect and good outcomes they worked for.	−0.02	−0.17	−0.29	0.46	0.18	0.59
My failures and successes are the direct result of my own behavior.	−0.14	−0.22	−0.21	0.45	0.10	0.55
I have absolute control over what happens to me in my life.	−0.31	0.01	−0.16	0.44	−0.15	0.59
People do not realize how much they can personally determine their own outcomes in life.	−0.12	−0.13	−0.20	0.34	0.10	0.43
People’s misfortunes are the result of the mistakes they make.	−0.10	−0.19	−0.12	0.31	0.02	0.53
My actions have a direct impact on things that happen to me.	−0.12	−0.22	−0.30	0.30	0.06	0.57
There’s no sense in taking a lot of safety precautions, since what is going to happen will happen no matter what you do.	0.25	0.22	0.40	−0.12	0.47	0.73
Life and death are entirely out of our control.	0.33	−0.09	0.03	0.03	0.46	0.63
If an accident is going to happen, there’s nothing you can do to prevent it.	0.32	0.07	0.19	−0.18	0.46	0.59
Life is written in a way that cannot be changed.	0.62	0.14	0.34	−0.28	0.43	0.78
Quite often one finds that what happens to people has no relation to what they do. What happens just happens.	0.26	0.26	0.37	−0.17	0.42	0.64
People who accept their circumstances in life are happier than those who try to change things.	0.13	0.13	0.19	0.03	0.38	0.53
People should focus on the present, since the future is completely out of our hands.	0.27	0.10	0.27	−0.08	0.38	0.59
A person’s future is determined the moment he or she is born.	0.61	0.14	0.34	−0.23	0.37	0.72
The secret of happiness is not to expect too much out of life, and to be content with what life brings you.	0.22	0.16	0.25	−0.05	0.36	0.56
People are happier when they don’t think about the future too much.	0.10	0.25	0.27	−0.13	0.33	0.49
If I am destined to stay healthy, I will stay healthy.	0.46	0.22	0.25	−0.13	0.26	0.58
When something bad happens, I accept it as unavoidable.	0.08	0.11	0.21	−0.02	0.26	0.50
I think that problems are solved on their own if you don’t worry about them.	0.18	0.25	0.38	−0.19	0.11	0.57

First items with bolded loadings are the items retained for the fatalism measure

#### Normality of Data

For the confirmatory factor analysis, normality for the data was assessed by analyzing the ratio of the skewness statistic and the standard error of the skewness per item (Curran et al. 1996; West et al. 1995). There were 23 skewed items, so since our data were not normally distributed, the diagonally weighted least-squares (DWLS) method was used to analyze the models for this study. The DWLS method outperforms the weighted least-squares (WLS) when sample sizes are limited (Flora and Curran 2004).

#### Confirmatory Factor Analysis

A sample of 391 participants was used to cross-validate the factor structure of the fatalism measure. The confirmatory factor analysis included five factors and each factor included six items, for a total of 30 items (Table 2). In order to calculate the model, the variance of the item with the highest loading per factor had to be constrained to one. Model fit was evaluated with the following results: The SBχ^2^ with 395° freedom was equal to 647.41 (p < 0.01). The rest of the values were: NNFI = 0.97, RMSEA = 0.04, CFI = 0.97, NFI = 0.94, GFI = 0.96, and SRMR = 0.05. Using Hu and Bentler (1999) criteria, all indices indicate a good model fit except for the SBχ^2^. Loadings for this model ranged from 0.37 to 0.87 (Table 3).

**Table 2:** Items selected for the multidimensional fatalism scale in english and spanish

Items	
Divine control 1. Everything that happens is part of God’s plan. 2. Everything that happens to a person was planned by God. 3. Whatever happens to me in my life, it is because that is the way God wanted it to happen. 4. God controls everything good and bad that happens to a person. 5. God has a plan for each person, and you cannot change his plan. 6. No matter how much effort I invest into doing things, at the end, God’s decisions will prevail.	Control divino 1. Todo lo que sucede es parte del plan de Dios. 2. Todo lo que le pasa a una persona fue planeado por Dios. 3. Cualquier cosa que me pase en la vida, es porque así quería Dios que pasara. 4. Dios controla todo lo bueno y lo malo que le sucede a una persona. 5. Dios tiene un plan para cada persona, y usted no puede cambiar su plan. 6. Por mucho esfuerzo que yo invierta en hacer las cosas, al final, la decisión de Dios prevalecerá.--
Luck 1. When I get what I want, it’s usually because I’m lucky. 2. How successful people are in their job is related to how lucky they are. 3. Some people are simply born being lucky. 4. When good things happen to people, it is because of good luck. 5. The really good things that happen to me are mostly because of luck. 6. Luck does not exist	Suerte 1. Cuando obtengo lo que quiero es usualmente es porque tengo suerte. 2. El grado de éxito que tienen las personas en su trabajo está relacionado con la cantidad de suerte que tienen. 3. Alguna gente simplemente nace siendo suertuda. 4. Cuando le pasan cosas buenas a la gente, es por buena suerte. 5. Las cosas realmente buenas que me pasan son generalmente por suerte. 6. No existe la suerte.
Helplessness 1. I feel that nothing I can do will change things. 2. Sometimes I feel there is nothing to look forward to in the future. 3. I feel that I do not have any control over the things that happen to me. 4. No matter how hard I try, I still cannot succeed in life. 5. I often feel overwhelmed with problems, since I do not have any control over solving these problems. 6. There’s nothing I can do to succeed in life, since one’s level of success is determined when one is born.	Desesperanza 1. Siento que nada de lo que yo pueda hacer cambiará las cosas. 2. A veces siento que no hay nada que esperar del futuro. 3. Yo siento que no tengo ningún control sobre las cosas que me pasan. 4. No importa qué tanto me esfuerce, todavía no puedo triunfar en la vida. 5. Con frecuencia me siento abrumado con problemas, ya que no tengo ningún control sobre la resolución de estos problemas. 6. No hay nada que yo pueda hacer para tener éxito en la vida, pues el nivel de éxito está determinado cuando uno nace.
Internality 1. I feel that when good things happen, they happen as a result of my own efforts. 2. What happens to me in the future mostly depends on me. 3. My life is determined by my own actions. 4. What people get out of life is always due to the amount of effort they put into it. 5. What happens to me is a consequence of what I do. 6. I can do almost anything if I really want to do it.	Internalidad 1. Siento que cuando pasan cosas buenas, suceden como resultado de mis propios esfuerzos. 2. Lo que me pase a mí en el futuro depende mayormente de mí. 3. Mi vida está determinada por mis propias acciones. 4. Lo que la gente obtiene de la vida es siempre debido a la cantidad de esfuerzo que le dedican. 5. Lo que me pasa a mí es consecuencia de lo yo haga. 6. Puedo hacer casi cualquier cosa si realmente quiero hacerlo.
Fatalism 1. I have learned that what is going to happen will happen. 2. If something bad is going to happen to me, it will happen no matter what I do. 3. If bad things happen, it is because they were meant to happen. 4. There is no sense in planning a lot; if something good is going to happen, it will. 5. Life is very unpredictable, and there is nothing one can do to change the future. 6. People die when it is their time to die and there is not much that can be done about it.	Fatalismo 1. He aprendido que lo que tiene que pasar, pasará. 2. Si algo malo me va a pasar, pasará sin importar lo que haga. 3. Si pasan cosas malas, es porque así tenían que pasar. 4. No tiene sentido hacer muchos planes; si algo bueno va a pasar, pasará. 5. La vida es muy imprevisible, y no hay nada que uno pueda hacer para cambiar el futuro. 6. La gente se muere cuando es su tiempo de morir y no hay mucho que se pueda hacer al respecto.

**Table 3:** Factor loadings and uniquenesses of confirmatory factor analysis and model with all parameters constrained to equality

Item	Factor loadings					Uniqueness
	Fatalism	Helplessness	Internality	Luck	Divine control	
FAT1	0.74 (0.72)					0.45 (0.49)
FAT2	0.37 (0.38)					0.86 (0.85)
FAT3	0.69 (0.72)					0.53 (0.48)
FAT4	0.66 (0.62)					0.56 (0.62)
FAT5	0.50 (0.50)					0.75 (0.75)
FAT6	0.62 (0.65)					0.61 (0.58)
HEL1		0.52 (0.57)				0.73 (0.67)
HEL2		0.71 (0.74)				0.49 (0.45)
HEL3		0.74 (0.75)				0.46 (0.44)
HEL4		0.53 (0.55)				0.72 (0.69)
HEL5		0.54 (0.57)				0.71 (0.67)
HEL6		0.41 (0.48)				0.83 (0.77)
INT1			0.69 (0.71)			0.52 (0.50)
INT2			0.62 (0.68)			0.61 (0.54)
INT3			0.62 (0.60)			0.61 (0.64)
INT4			0.56 (0.55)			0.69 (0.70)
INT5			0.63 (0.61)			0.61 (0.63)
INT6			0.64 (0.61)			0.60 (0.62)
LUC1				0.71 (0.73)		0.50 (0.47)
LUC2				0.69 (0.67)		0.52 (0.56)
LUC3				0.73 (0.77)		0.47 (0.40)
LUC4				0.42 (0.48)		0.82 (0.77)
LUC5				0.71 (0.74)		0.50 (0.45)
LUC6				0.72 (0.72)		0.48 (0.49)
DIV1					0.82 (0.86)	0.32 (0.26)
DIV2					0.87 (0.87)	0.24 (0.23)
DIV3					0.72 (0.76)	0.48 (0.43)
DIV4					0.85 (0.86)	0.28 (0.26)
DIV5					0.79 (0.80)	0.37 (0.36)
DIV6					0.79 (0.80)	0.38 (0.35)

Values in parenthesis are values of the model with all parameters constrained to equality. *FAT* Fatalism, *HEL* Helplessness, *INT* Internality, *LUC* Luck, *DIV* Divine control

The factors were intercorrelated (see Table 4). Cronbach’s alpha coefficients (α) indicating internal consistency of the scales across samples were acceptable for all subscales: fatalism (α = 0.76), helplessness (α = 0.76), internality (α = 0.80), luck (α = 0.82), divine control (α = 0.92). Some shrinkage was observed upon cross-validation, as expected.

**Table 4:** Correlation between factors measured with the multidimensional fatalism measure

Factors	Ineluctable destiny	Helplessness	Internality	Luck	Divine control
Ineluctable destiny	–				
Helplessness	41	–			
Internality	−0.15	−0.36	–		
Luck	0.25	0.31	−0.18	–	
Divine control	0.50	0.18	−0.21	0.10	–

All correlations were significant at *p* < 0.01

#### Test-Retest Reliability

One week test-retest reliability was assessed by correlating factors scores derived from the first session with the factor scores from the second session. Five hundred seventy-eight participants completed the fatalism form for Time 2. The Pearson product–moment correlation for the fatalism factor was *r* = 0.71, for helplessness it was *r* = 0.73, for internality it was *r* = 0.63, for luck it was *r* = 0.77, and for divine control it was *r* = 0.87.

### Measurement Invariance

Because the *χ*^2^ and the Δχ^2^ indices are sensitive to sample size and to non-normal data (Cheung and Rensvold 2002; Little 1997; Marsh et al. 1997), we used the difference in the comparative fit index (ΔCFI) as an index to assess invariance between nested models and the baseline model. A ΔCFI of less than or equal to 0.01 suggests similar model fit (Byrne et al. 2007; Cheung and Rensvold 2002).

Since our confirmatory factor analysis indicated a good model fit, measurement invariance was assessed across English and Spanish. A confirmatory factor analysis was done for each of the samples. Both the English and Spanish models had good fit indices (Table 5). Thus, configural invariance was analyzed. The configural invariance model is the baseline model to which the rest of the nested models were compared using the ΔCFI value (Byrne et al. 2007; Cheung and Rensvold 2002). The configural invariance model showed good fit (Table 5), so loadings were constrained to equality across samples (metric invariance). The model fit well and the ΔCFI value was less than 0.01 (Table 5). Next, item intercepts were constrained to equality (scalar invariance), and good fit was once again observed (Table 5). Finally, the item errors were constrained to equality (strict factorial invariance). The ΔCFI value was less than 0.01 and all of the fit indices (Table 5) were in the range recommended by Hu and Bentler (1999). To test structural invariance, factor variances and covariances were constrained to equality across language. Again, the model was a good fit and the ΔCFI value was less than 0.01 (Table 5).

**Table 5:** Fit indices for nested models across english and spanish fatalism forms

Model	SBχ^2^	df	ΔSBχ^2 a^	NNFI	RMSEA	CFI	ΔCFI ^a^	NFI	GFI	SRMR
Confirmatory factor analysis (English sample)	728.88**	395	–	0.97	0.04	0.9762	–	0.95	0.96	0.05
Confirmatory factor analysis (Spanish sample)	538.88**	395	–	0.98	0.04	0.9807	–	0.93	0.95	0.06
1. Configural invariance (Number of factors and items in same factors)	1255.93**	790	–	0.98	0.04	0.9782	–	0.94	0.95	0.06
2. Metric invariance (Loadings)	1276.29**	815	23.13	0.98	0.04	0.9784	−0.0002	0.94	0.95	0.07
3. Scalar invariance (Intercepts)	1368.70**	845	113.04**	0.97	0.04	0.9755	0.0027	0.94	0.94	0.07
4. Strictc factorial invariance (error/uniqueness)	1425.69**	875	165.81**	0.97	0.04	0.9743	0.0039	0.94	0.94	0.08
5. Structural invariance (Factor variances and covariances)	1454.55**	890	196.88**	0.97	0.04	0.9736	0.0046	0.93	0.93	0.09
5. Structural invariance—evaluation of factor means	1397.46**	885	142.70**	0.98	0.04	0.9760	0.0022	0.94	0.94	0.08

*SBχ* ^*2*^ Satorra-Bentler *χ* ^2^, *df* degrees of freedom, *ΔSBχ* ^*2*^ difference in SBχ^2^ between baseline model (configural invariance) and nested models, *NNFI* non-normed fit index, *RMSEA* root mean square error of approximation, *CFI* comparative fit index, *ΔCFI* difference in CFI between baseline model (configural invariance) and nested models, *NFI* norm fit index, *GFI* goodness of fit index, *SRMR* standardized root mean squared residual

– value not calculated

^a^Nested models were compared to Model 1

** *p* < 0.01

Since the scale had strict configural invariance and structural invariance in its factor variances and covariances, it was possible to calculate differences in factor means across the English and Spanish samples. Following Byrne (1998), the factor means of the English sample were permitted to vary and the factor means of the Spanish sample were fixed at zero. Item intercepts in the Spanish sample were also held invariant (Byrne 1998). The analysis indicated significant differences in the fatalism factor, the internality factor, the luck factor, and the divine control factor and the difference estimates were 0.20, −0.14, 0.06, and 0.08, respectively. Even though the differences are significant, each difference is less than a point of the scale.

### Convergent and Discriminant Validity

To assess convergent and discriminant validity, the fatalism factors were correlated with the selected criterion measures. Table 6 shows the correlation between the fatalism factors and measures of optimism, internality, belief in good luck, depression, and religiosity. As predicted, the helplessness factor had its highest correlations with optimism and with depression, the luck factor had its highest correlation with belief in good luck, the divine control factor had the highest correlation with religiosity, and the internality factor had its highest correlation with the internality dimension of the ASQ. The correlations with the ASQ were low, probably due in part to the low internal reliability of the criterion measure in this sample (α = 0.36). Discriminant validity was evidenced by lower correlations between the scales and criterion variables with which associations were not hypothesized. The fatalism factor was not expected to correlate highly with any of these measures, though it correlated significantly with all of them, most highly with depression.

**Table 6:** Correlation between factors of the multidimensional fatalism measure, internal reliability, and assessment of convergent and discriminant validity

Factors	FA	HE	IN	LU	DC	LOT-R	ASQ	BIGL	PHQ-9	DRI
Fatalism (FA)	0.76					−0.17**	−0.10**	0.11**	0.20**	0.15**
Helplessness (HE)	0.41**	0.76				−0.49**	−0.09*	0.02	0.40**	−0.04
Internality (IN)	−0.15**	−0.36**	0.80			0.14**	0.24**	0.01	−0.06	−0.09*
Luck (LU)	0.25**	0.31**	−0.18**	0.82		−0.09*	−0.10**	0.60**	0.05	−0.11**
Divine control (DC)	0.50**	0.18**	−0.21**	0.10**	0.92	0.06	−0.05	0.05	−0.01	0.57**

*LOT-R* Life Orientation Test— Revised (optimism), *ASQ* Attributional Style Questionnaire— internality scale, *BIGL* Belief in Good Luck Scale, *PHQ*-*9* Patient Health Questionnaire (depression), *DRI* Duke Religion Index (religiosity)

Correlations in bold are the correlations expected to reflect convergent validity

Values in diagonal and italics are internal consistencies of each factor measured by Cronbach’s alpha

* *p* < 0.05

** *p* < 0.01

Finally, the associations between socioeconomic status, acculturation, and the fatalism factors were tested. Socioeconomic status did significantly correlate with the fatalism factors. The only factor to be correlated with acculturation was internality (*r* = −0.13, *p* < 0.01 with the Mexican Orientation Scale and *r* = 0.12, *p* < 0.01 with the Anglo Orientation Scale).

## Discussion

The factor structure of the study done by Esparza (2005) was well-replicated in our study with items written specifically for that purpose. Although some items had shared loadings, especially those on the fatalism factor, there were enough items per factor to choose those that would provide distinct measure of the constructs in question. Six items were selected for each subscale to balance concision with internal reliability. The confirmatory factor analysis cross-validated the structure of the fatalism measure in an independent sample, and there was relatively little shrinkage in internal consistency upon cross-validation. One-week test-retest reliabilities were acceptable, except for the internality factor, which had an *r* = 0.63.

The multidimensional fatalism measure developed has the potential of bringing unity in the measurement of fatalism and clarity in the findings. The developed scale is a reliable and valid measure with good psychometric properties that can be used interchangeably in English or in Spanish.

It seems clear that the first factor encompasses the core of the fatalism construct as defined in the modern lexicon. It reflects a tendency to view all events (whether positive or negative) as fixed in advance and inevitable, while not necessarily anticipating a particular outcome.

The second factor to emerge, labeled “helplessness,” is similar in content to what Acevedo (2005a) refers to as structural fatalism. It reflects a pervasive pessimistic outlook and is strongly linked to depression. However, in psychology this construct has its own rich literature separate from work on fatalism (e.g., Seligman 1975) and has been most frequently studied in the context of psychopathology, rather than normal personality. It may be best to distinguish it from fatalism in future research.

The divine control factor is similar to the concept of cosmological fatalism that Acevedo proposes, and also reflects secondary control expectations, as defined by Morling and Evered (2006). However, this factor was the least well correlated with the others, and virtually uncorrelated with helplessness and internality. Content of the items, while reflecting a strong belief in divine intervention, did not generally make reference to predetermination of events. The strong internal consistency of the factor suggests that belief in divine control is an important variable in its own right, and the present results suggest that it should be measured separately from fatalism. Indeed, work such as that by Wallston et al. (1999) has advanced the study of divine control beliefs.

Even further removed from the core construct of fatalism are the factors of luck and internality. The existing conceptualizations of luck in the psychological literature have ranged from that of a random and unpredictable variable (e.g., Weiner 1974) to a relatively stable individual difference (e.g., Darke and Freedman 1997; Wagenaar and Keren 1988). Items loading on the current luck factor reflect these two extremes, from “To a great extent my life is controlled by accidental happenings,” to “Some people are just born lucky.” However, again this literature has developed independently of work on fatalism (e.g., Pritchard and Smith 2004) and may be best identified as a separate domain.

As for internality, it is perhaps surprising that this factor emerged distinctly from the other four factors and had the lowest correlation with the core fatalism factor. Multiple researchers in the past have assumed that low internal locus of control is indicative of high fatalism (Cole et al. 1978; De Brabander, et al. 1999; Joiner, et al. 2001). However, the present results suggest that the two concepts do not necessarily define opposite ends of a single dimension.

Of the five factors proposed by Esparza (2005), the first and largest factor is the only one that is not already well established under a different name in the “nomological net” of psychological constructs, with a corresponding supportive literature. We believe that this reflects the fact that this factor best defines the concept of “fatalism,” and should be the primary target of future fatalism research. It is essential that researchers define more clearly the nature of the variable they wish to study when conducting work on fatalism. Indeed, it is possible that diversity in previous measures of fatalism might help explain the inconsistency across existing studies of the construct.

This is the first report of measurement invariance data from an instrument developed simultaneously in two languages. Following the guidelines by Tanzer (2005), simultaneous development in English and Spanish resulted in a decentered instrument that is invariant across language in its factor structure, loadings, item intercepts, item uniqueness, and factor variances and covariances. Indeed, the highly consistent results of the language comparisons are atypical in the measurement invariance literature, lending support to the simultaneous development approach.

Once measurement invariance was established and latent mean differences were evaluated, there were significant differences between the English and Spanish samples on all of the factors, except for helplessness. However, although the differences were statistically significant, none of them exceeded one point on a scale from 6 to 30. The greatest difference (on the fatalism factor) was two-tenths of one point, there does not appear to be a practically significant effect of language on latent means.

The convergent and discriminant analyses shows how the five related but distinct factors behave differently with other constructs. Each factor, except for the internality factor, correlated strongly and uniquely with its designated construct in the hypothesized manner. However, other correlations varied not only in magnitude, but also in direction, depending on the factor under consideration. For example, while religiosity correlated positively with divine control and fatalism, it was negatively associated with the luck factor. Past studies in which these factors have been conflated in a single fatalism measure have been unable to detect such subtleties.

Although the majority of the current sample was Hispanic (86.2 %) this may not have greatly impacted the factor structure identified. Cole et al. (1978), compared fatalistic attitudes of Mexicans and Chicanos with participants from the US, Ireland, and West Germany, and found no difference in fatalism indicators. Future directions include analyzing the factor structure of the fatalism measure in other populations that are not college students, with other ethnic groups, with different age groups, in different parts of the country or other countries.

One limitation of this study is that there was no control for Type 1 error since there were numerous analyses done with the data. While explicit directional hypotheses were stated in most cases, there remains the possibility that some bivariate associations in the current study may have been exaggerated due to unique sample characteristics. Also, due to concern about overburdening student participants, no health measures could be included in this study to analyze their relationships with the fatalism factors. For future studies, another internality instrument should be used to compare it to the internality factor of the scale. Even though the highest correlation of the internality factor was with the ASQ, the correlation was not strong (*r* = 0.24) since the internality dimension of the ASQ had a poor internal consistency (α = 0.36).

The present study contributes to the clarification of the constructs that have been assessed in past research on fatalism. The new measure of fatalism and related constructs may further advance understanding in the domain. We propose to use the fatalism factor of this measure to analyze the relationship between fatalism and other behaviors. The other four factors (helplessness, internality, luck, and divine control), even though some have used to measure fatalism, they should be analyzed separately and not to be confused with fatalism. For example, a study reports a significant correlation of *r* = 0.19 between fatalism and a cardio-metabolic dysfunction score (Espinosa and Gallo 2013). They use a fatalism measure from Cuéllar et al. (1995a)b that include the following items: “People can’t really do much to change what happens in life. You just have to accept things”, “It’s more important to enjoy life now than to plan for the future”, “I live for today because I don’t know what will happen in the future”, “I don’t plan ahead because most things in life are a matter of luck”. According to our findings, the only item that reflects the construct of fatalism is the first one, but in the Cuéllar fatalism measure all of these items are included in their fatalism factor. If the same analysis was done using our scale, then the relationship between fatalism and cardio-metabolic dysfunction would be more precise. Also the other four factors of our scale could be correlated to analyze those relationships, which are different from fatalism.

This measure may be used to test theoretical associations between the proposed constructs and important health behaviors. There are several studies in which fatalism has been related to health behaviors. Some examples include cardio-metabolic dysfunction (Espinosa and Gallo 2013), cancer screening (Espinosa and Gallo 2011), HIV and AIDS prevention (Gwandure and Mayekiso 2012), protective sun behaviors (Coups et al. 2014), and adherence to highly active antiretroviral therapy (Vyas et al. 2014). We expect to have more exact results using this scale in health related behavior studies since it has a factor that is purported to measure specifically the fatalism factor without having items that represent other constructs like luck, helplessness, divine control or internality.
